# Intracranial hypertension as the primary symptom of gastric signet-ring cell carcinoma: A case report and literature review

**DOI:** 10.1097/MD.0000000000004687

**Published:** 2016-09-02

**Authors:** Jiali Pu, Lingjia Xu, Xinzhen Yin, Baorong Zhang

**Affiliations:** Department of Neurology, 2nd Affiliated Hospital, School of Medicine, Zhejiang University, Hangzhou, Zhejiang, People's Republic of China.

**Keywords:** case report, gastric signet-ring cell carcinoma, intracranial hypertension, subcutaneous metastases

## Abstract

**Background:**

Intracranial hypertension (IH) is a neurological disorder characterized by increased intracranial pressure. It is a poorly understood syndrome that most commonly manifests nonspecific symptoms such as stroke-like headache, vision changes, nausea, vomiting, and papilledema. IH has been reported in young cancer patients but never in association with gastric signet-ring cell carcinoma.

**Methods:**

Here, we discuss the case of an 18-year-old girl with gastric signet-ring cell carcinoma in which IH was the primary symptom accompanied by the even rarer symptom of cutaneous metastases. We also present a review of the relevant literature. The patient experienced frequent headaches, vomiting, and blurred vision but showed no abnormal findings on cranial imaging studies. Further examination showed multiple skin nodules on the abdomen. Then pathological and immunohistochemical examination of gastroscopic specimens and the biopsied subcutaneous nodules were done.

**Results:**

Pathological and immunohistochemical examination of gastroscopic specimens and the biopsied subcutaneous nodules confirmed gastric signet-ring cell carcinoma with skin metastases.

**Conclusion:**

To our knowledge, this is the first reported case of gastric signet-ring cell carcinoma primarily presenting IH and accompanied by subcutaneous metastases. This case emphasizes the importance of excluding malignancy from the differential diagnosis of IH.

## Abstract 2

Supplemental Digital Content is available in the text

## 1 Introduction

Intracranial hypertension (IH) is a neurological disorder characterized by increased intracranial pressure. It is a poorly understood syndrome that most commonly presents as stroke-like headache, vision changes, nausea, vomiting, and papilledema.^[1–3]^ As it sometimes progresses to cerebral hernia crisis leading to respiratory and circulatory failure and even death, early diagnosis and appropriate treatment of IH is critical. IH has been reported in cancer patients^[4,5]^ but never in association with gastric signet-ring cell carcinoma. Further, gastrointestinal tumors presenting neurological problems are especially rare,^[6]^ and diagnosis in such cases is often delayed because of the nonspecific symptoms.

Here, we report the case of an 18-year-old girl whose main symptoms were IH and subcutaneous nodules in the abdomen. Pathological and immunohistochemical reports of gastroscopic specimens and biopsy examination of the subcutaneous nodules confirmed gastric signet-ring cell carcinoma with skin metastases. To our knowledge, this is the first case of gastric signet-ring cell carcinoma primarily presenting IH and accompanied by subcutaneous metastases.

## 2 Case report

An 18-year-old girl was admitted to the emergency room with frequent headaches, vomiting, and blurred vision that had persisted 2 months. She had also had low-grade fever (37.0–38.0 °C) for the previous 15 days. On neurological examination, she was fully alert and suspected neck rigidity, and the remainder of neurological examination was normal.

**Figure 1:**
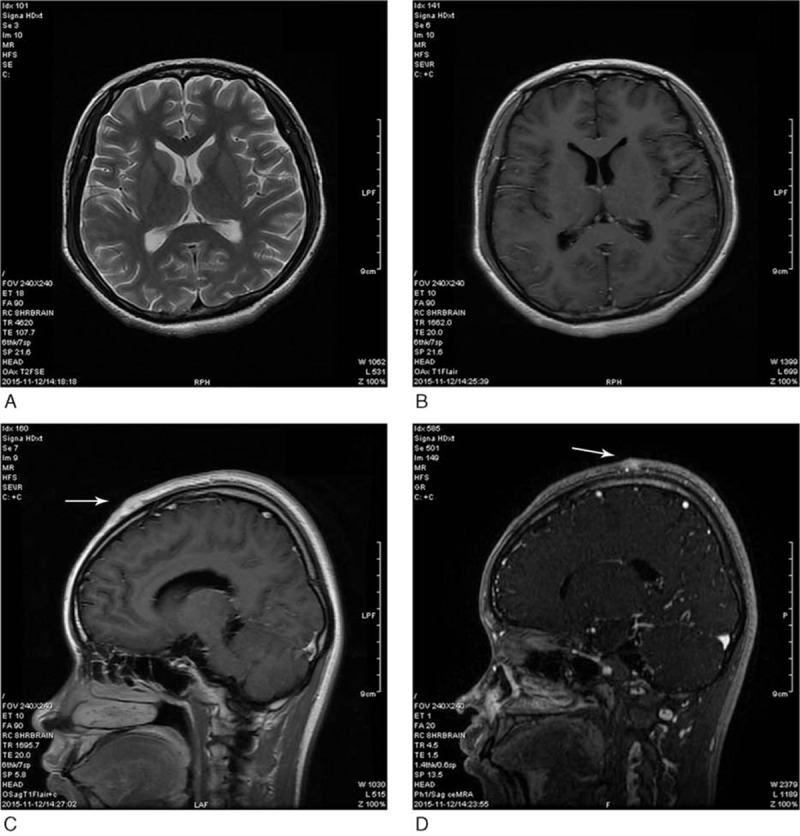
Cranial magnetic resonance imaging scans (A, B, C, D) showing no abnormalities. The arrows show a suspicious enhanced signal in the frontal and parieto-occipital regions.

At the local hospital, she underwent cranial and lung computed tomography (CT) scans as well as gastroscopic examination, none of which showed any obvious abnormalities. Cerebrospinal fluid (CSF) was first examined at the emergency department, and the results were normal except that the pressure was high (>400 mm H_2_O). Cranial magnetic resonance imaging (MRI) conducted at our hospital showed no obvious abnormalities either, although a suspicious enhanced signal was noted at the frontal and parieto-occipital regions of the scalp on both sides (Fig. 1). Therefore, the patient was hospitalized with a diagnosis of IH.

On the further physical examination, multiple skin nodules were found on the abdomen. She had a family history of esophageal cancer (her grandfather and granduncle died of esophageal cancer). The results of serum tumor marker examination showed that both carcinoembryonic antigen (12.2 ng/mL) and cancer antigen 125 (43.7 U/mL) levels were mildly to moderately elevated. Other routine laboratory tests were normal (Supplementary Table 1). Abdominal CT showed multiple lymph nodes behind the peritoneum. A biopsy specimen was obtained from a subcutaneous nodule in the abdomen.

**Figure 2:**
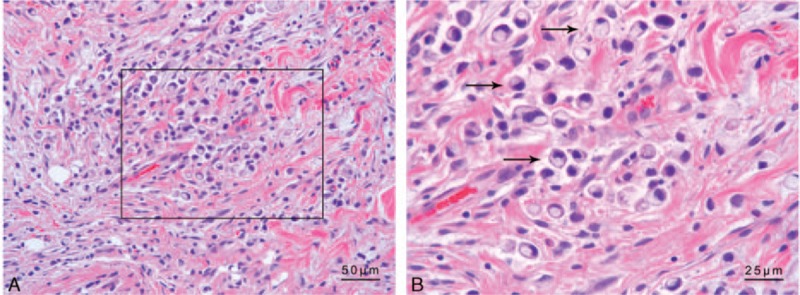
Pathological examination of a biopsy specimen of the subcutaneous abdominal nodules. Sections from the biopsied tissue were examined using hematoxylin and eosin staining. The results showed infiltration of a large number of signet-ring cells. The arrows show typical signet-ring cells. The nucleus is stained blue. (A) 200×. (B) 400×.

**Figure 3:**
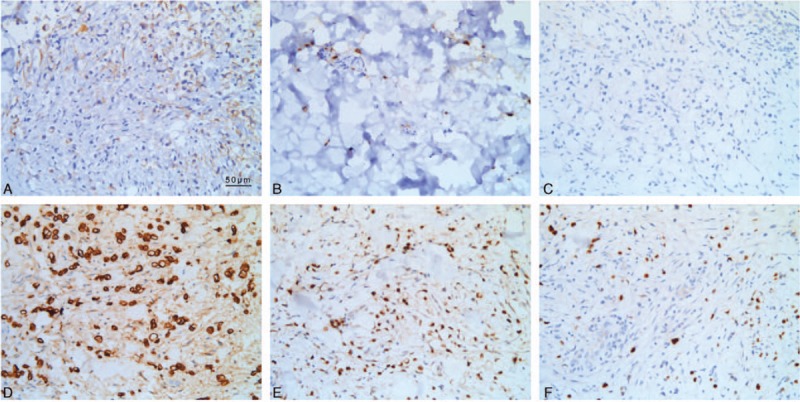
Immunohistochemical examination of a biopsy specimen of the subcutaneous abdominal nodules. Sections from the biopsied tissue were immunohistochemically examined with various antibodies as follows. Positive staining (brown) was seen in the cytoplasm or nucleus, and positive immunoreactivity was detected for Muc1 (A), Muc2 (B), CK20 (D), p53 (E), and Ki-67 (F). CK7 (C) was not detected. Scale bars: 50 μm.

Follow-up examination showed that the patient's headaches and visual complaints had progressively worsened, so we repeated lumbar puncture and CSF examination. At this time, the intracranial pressure was still very high (>400 mm H_2_O); further, the glucose content of the CSF was mildly elevated (4.50 mmol/L). After the second lumbar puncture, we increased the dosage of dehydrating agents, from 250 mL of glycerol fructose sodium chloride ivgtt Q12H and 125 mL mannitol ivgtt Q8H to 250 mL of glycerol fructose sodium chloride ivgtt Q12H and 125 mL mannitol ivgtt Q6H. Other treatments included agents for protecting the gastric mucosa and nutrition supplements. However the pathological results of the biopsy specimen showed invasive growth of malignant signet-ring cells (Fig. 2). The results of immunohistochemical examination showed that she tested negative for CK7, synaptophysin (Syn), chromogranin A (CgA), Muc5AC, human epidermal growth factor receptor-2 (HER2), and Muc6; positive for CK20, Muc1, Muc2, E-cadherin, and p53; the Ki-67 index was about 87% (Fig. 3, Supplementary Table 2). To better understand the origin of the tumor, gastroscopic examination was performed, and the results showed multiple nodular hyperplasia accompanied by gastric ulcer. Pathological examination showed chronic mucosal inflammation in the big bend of the gastric antrum with small, focal signet-ring cells, confirming the diagnosis of signet-ring cell carcinoma. We also tested the autoimmune antibody series and paraneoplastic syndrome-related antibody in the CSF to determine the causes of the neurological symptoms, but both tests yielded negative results. The results of clinical laboratory examinations are given in Supplementary Table 1. The patient's prognosis was poor and her parents decided to give up treatment, choosing to return to their hometown the day after the gastroscopy.

This work was approved by the Department of Medicine of the Second Affiliated Hospital, Zhejiang University and the informed consent was obtained.

## 3 Discussion

Signet-ring cell carcinoma is a rare histological variant of mucinous adenocarcinoma and usually is associated with a poor prognosis, because it is poorly differentiated.^[7]^

Signet-ring cells are characterized by a slightly curved nucleus found near the cell membrane and a mucin-filled vacuole that occupies most of the cell. The mucin appears clear in stains, but it is diastase resistant, periodic acid-Schiff and mucicarmine positive.^[8,9]^ In Goldstein et al's^[10]^ study on 27 cases of gastric signet-ring cell carcinoma, the expression status of CK7/20 was very similar to that in the present study, and this previous study suggested that the CK7/20 pattern is useful in determining the primary and metastatic origin of this carcinoma. The present study confirmed the importance of the CK7/20 pattern in primary gastric signet-ring cell carcinoma. p53 is a product of the well-known tumor suppresser gene *p53*,^[11]^ and this nuclear protein is thought to control the cell cycle, apoptosis, and maintenance of genomic stability. *p53* gene mutations are the commonest genetic alteration in human tumors, and they indicate the potential malignancy of tumors.^[12]^ The Ki-67 antigen is a nuclear nonhistone protein expressed by cells in the G1, G2, M, and S phases. Therefore, it is a valuable indicator in the analysis of cell proliferation.^[12,13]^ Both Syn and CgA are markers of neuroendocrine cells and are useful in establishing the diagnosis of neuroendocrine tumors.^[14,15]^ Our findings ruled out the possibility of gastrinoma in the present case. E-cadherin helps maintain cell polarity and characteristics, inhibits cell growth for regulation, and suppresses cancer invasion.^[16]^ Abnormal E-cadherin expression often indicates advanced gastric cancer with a poor prognosis.^[16]^ Human epidermal growth factor receptor-2 (HER2) is a member of the EGFR family, breast cancer patients who test positive for this receptor are generally expected to have a poor prognosis.^[17,18]^ Overexpression of HER2 in patients with gastric cancer ranges from 6% to 23%,^[19,20]^ and its prognostic significance in gastric cancer is controversial.^[21]^ The immunohistochemical results obtained in the present study confirmed that the nature of gastric cancer in this case was signet-ring cell carcinoma.

Gastric signet-ring cell carcinoma has been known to present many different clinical symptoms, including cutaneous metastases,^[22,23]^ but primary symptoms involving the central nervous system are rare.^[24]^ Erdoğan et al^[5]^ reported a case of IH due to duodenal signet-ring cell tumor, but it was not accompanied by cutaneous metastases. Lee et al^[25]^ reported a patient with gastric signet-ring cell tumor showing leptomeningeal involvement. Table 1 summarizes reported cases of gastric carcinoma in which neurological symptoms were presented initially. To our knowledge, this is the first case of gastric signet-ring cell carcinoma primarily presenting IH and accompanied by cutaneous metastases.

**Table 1:**
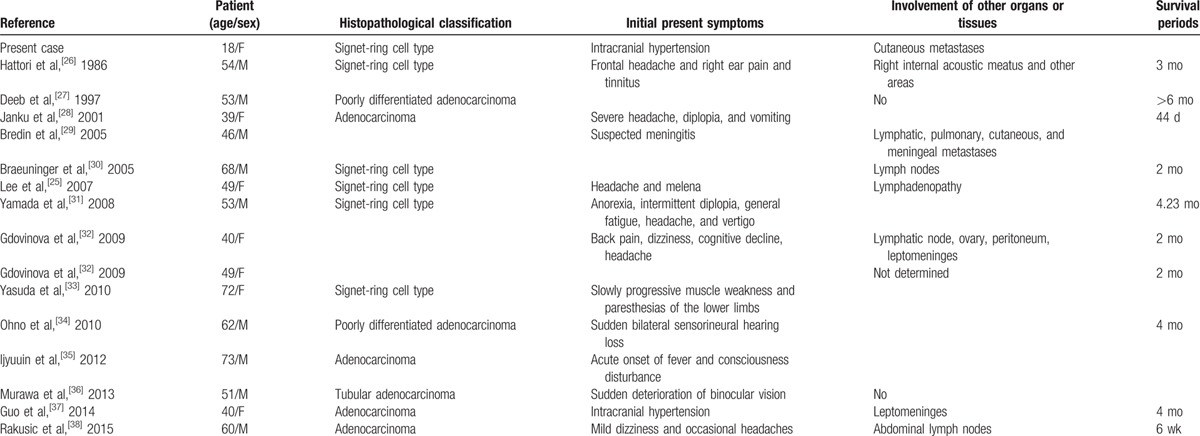
Reported cases of gastric carcinoma that initially presented with neurological problems.

The pathophysiology of IH remains poorly explained, although various mechanisms have been proposed. The first mechanism is related to the volume compensation that occurs in the cranium when an intracranial mass occurs. The cranium is a rigid structure and mainly contains the brain (80%), blood (10%), and CSF (10%). When an intracranial mass appears, a compensatory change in volume must occur through a reciprocal decrease in venous blood or CSF to keep the total intracranial volume constant. However when the mass is very large and cannot be compensated for in this manner, IH occurs. This may be the most common cause of IH. Second, hypersecretion and improper drainage of CSF may cause IH. The choroid plexus is responsible for CSF secretion,^[39,40]^ and hypersecretion is known to occur in cases of choroid plexus papilloma, a rare pediatric tumor.^[41]^ Further, the arachnoid granulations are believed to be responsible for CSF clearance,^[42]^ and subarachnoid hemorrhage or meningitis can result in communicating hydrocephalus, which may cause IH, since CSF absorption depends on the pressure gradient between the venous sinus and the subarachnoid space, and an increase in venous pressure warrants a concomitant increase in CSF pressure to maintain absorption rates.^[40,43,44]^ Third, increased cerebral arterial pressure and cerebral venous pressure lead to IH. Normally, intracranial pressure is maintained by cerebral arterial pressure, which itself is subject to cerebral autoregulation, because of which intracranial pressure remains constant over a wide range of systemic arterial blood pressures. If a tumor invades the arteries or capillaries, it may affect cerebral autoregulation and consequently lead to IH. Additionally, the increase in venous sinus pressure resulting from venous sinus stenosis is an obvious mechanism underlying IH.^[43,44]^ Lastly, cerebral edema, obesity, and pseudotumor cerebri could also underlie IH. Pseudotumor cerebri is defined as idiopathic or secondary IH with iatrogenic causes (antibiotics, hormonal factors, excess vitamin A, etc.) or caused by associated disorders (anemia, hormonal dysfunctions, respiratory dysfunction, etc.).^[43]^

Clinically, CT and MRI are useful methods for assessing the intracranial masses, hydrocephalus, and ventricular enlargement causes of IH. However, as mentioned above, cranial MRI showed no obvious abnormalities in the present case, and laboratory examinations also did not yield any clues regarding other possible causes of IH.

On the monism of medicine, we consider that the IH is related to the gastric signet-ring cell carcinoma preferentially. As signet-ring cells are a group of malignant cells that have naturally lost cell–cell adhesion properties and consequently present as individual cells or loose clusters,^[7]^ they tend to diffusely infiltrate the stroma and have a propensity toward vascular invasion, lymph node involvement, and distant widespread metastasis.^[45]^ However, the cranial images of our patient showed no such indications. Other causes of IH are meningeal carcinomatosis and paraneoplastic syndrome,^[6,46]^ but the latter was not identified on CSF testing in the present case. Additionally, cranial MRI showed a suspicious enhanced signal in the frontal and parieto-occipital scalp regions on both sides, but we could not confirm whether it was related to meningeal carcinomatosis.

In many situations, meningeal carcinomatosis does not appear in imaging studies, and it may have been the cause of IH in the present case. In addition, malignant carcinoma tends to induce the hypercoagulable state, leading to cerebral venous sinus stenosis,^[47,48]^ and this may have been the cause of IH in the present case. Magnetic resonance venography or cerebral digital subtraction angiography could have confirmed this possibility, but we did not perform these tests.

In summary, we presented the case of gastric signet-ring cell tumor presenting IH and cutaneous metastases in an 18-year-old girl. This case emphasizes the importance of excluding malignancy from the differential diagnosis of IH.

## Supplementary Material

Supplemental Digital Content
