# Supplementary material for: Intracranial hypertension as the primary symptom of gastric signet-ring cell carcinoma: A case report and literature review
Source: Medicine (Baltimore). 2016 Sep 2;95(35):e4687. doi: 10.1097/MD.0000000000004687 (PMC5008581; doi:10.1097/MD.0000000000004687)
Supplement: Supplemental Digital Content [file medi-95-e4687-s001.doc]

**Supplementary Table.1 The clinical laboratory examiniations of the patient**.

|  |  | Result | Normal value |  |
| --- | --- | --- | --- | --- |
| Tumor markers | Carcino-embryonic antigen | 12.2↑ | ＜5 | ng/mL |
|  | Alpha fetal protein | 2.1 | ＜20 | ng/mL |
|  | Cancer antigen 125 | 43.7↑ | ＜35 | U/mL |
|  | Cancer antigen 153 | 25.1 | ＜30 | U/mL |
|  | Cancer antigen 199 | 2.0 | ＜37 | U/mL |
|  | Cancer antigen 242 | 2.1 | ＜20 | U/mL |
|  | Cytokeratin211 | 0.9 | ＜5 | ng/mL |
|  | Neuronspecific enolase | 10.3 | ＜25 | ng/mL |
|  | Squamous cell carcinoma antigen | 0.4 | ＜1.5 | ng/mL |
|  | β-human chorionic gonadotropin | 1.2 | ＜5 | U/L |
| Autoimmune antibodies | Anti-nuclear antibodies | Negtive | Negtive |  |
|  | Rheumatoid factor | Negtive | Negtive |  |
|  | Antineutrophil cytoplasmic antibodies | Negtive | Negtive |  |
|  | Antiphospholipid antibodies | Negtive | Negtive |  |
|  | Complement | Negtive | Negtive |  |
| CSF test | Colour | Colourless | Colourless |  |
|  | Occult blood test | Negtive | Negtive |  |
|  | Karyocyte | 4 | ＜8 |  |
|  | Erythrocyte | 1 | ＜1 |  |
|  | Pandys test | Weekly positive | Negtive or weekly positive |  |
|  | Chlorine | 125.9 | 120-130 | mmol/L |
|  | Glucose | 4.50↑ | 2.2-3.0 | mmol/L |
|  | Adenosine deaminase | 1 |  | U/L |
|  | Protein | 27.20 | 8-43 | mg/dL |
|  | Bacteria smear and staining | Negtive | Negtive |  |
|  | Germiculture and drug sensitive test | Negtive | Negtive |  |
|  | Fungal culture and drug sensitive test | Negtive | Negtive |  |
|  | The tubercle bacillus culture and drug sensitive test | Negtive | Negtive |  |
| Other laboratory examinations | Blood routine test | Normal |  |  |
|  | Urine routine test | Normal |  |  |
|  | Facal routine test | Normal |  |  |
|  | Coagulation spectrum | Normal |  |  |
|  | Blood biochemical test | Normal |  |  |
|  | Glycosylated hemoglobin series | Normal |  |  |
|  | Myocardial enzyme spectrum | Normal |  |  |
|  | Immunoglobulin series | Normal |  |  |
|  | Thyroid hormones and antibodies | Negtive | Negtive |  |
|  | Other hormones | Normal |  |  |
|  | Vitamine B12,folic  acid and ferritin contents | Normal |  |  |
|  | Syphilis antibodies | Negtive | Negtive |  |
|  | HIV antibodies | Negtive | Negtive |  |
|  | T-spot test | Negtive | Negtive |  |
|  | Serumal fungus 1,3-β-D-glucan and glactomannan test | Negtive | Negtive |  |

Supplementary Table 2: Antibodies and their Dilutions

| Protein | Species of Antibody | Vendor (catalog#) | Dilution |
| --- | --- | --- | --- |
| CK7 | Mouse | ZSGB-BIO (ZM0071) | 1:300 |
| CK20 | Rabbit | ZSGB-BIO (ZA0574) | 1:200 |
| Syn | Rabbit | ZSGB-BIO (ZA0506) | 1:300 |
| CgA | Rabbit | ZSGB-BIO (ZA0507) | 1:200 |
| E-Cadherin | Mouse | ZSGB-BIO (ZM0092) | 1:50 |
| Ki-67 | Mouse | ZSGB-BIO (ZM0167) | 1:300 |
| p53 | Mouse | ZSGB-BIO (ZM0408) | 1:500 |
| Muc1 | Mouse | ZSGB-BIO (ZM0391) | 1:100 |
| Muc2 | Mouse | ZSGB-BIO (ZM0392) | 1:100 |
| Muc5AC | Mouse | ZSGB-BIO (ZM0395) | 1:100 |
| Muc6 | Mouse | ZSGB-BIO (ZM0396) | 1:100 |
| HER2 | Rabbit | VENTANA (F06908) | 1:500 |
